# Priority setting during the COVID-19 pandemic: going beyond vaccines

**DOI:** 10.1136/bmjgh-2020-004686

**Published:** 2021-01-18

**Authors:** Iestyn Williams, Beverley Essue, Elysee Nouvet, Lars Sandman, S Donya Razavi, Mariam Noorulhuda, Susan Goold, Marion Danis, Godfrey Biemba, Julia Abelson, Lydia Kapiriri

**Affiliations:** 1 Health Services Management Centre, University of Birmingham, Birmingham, UK; 2 Institute of Health Policy, Management and Evaluation, University of Toronto, Toronto, Ontario, Canada; 3 School of Health Studies, Western University, London, Ontario, Canada; 4 Department of Health, Medicine and Caring Sciences, Linköping University, Linkoping, Sweden; 5 Department of Health, Aging and Society, McMaster University, Hamilton, Ontario, Canada; 6 Department of Bioethics, National Institutes of Health, Bethesda, Maryland, USA; 7 Center for Bioethics and Social Sciences in Medicine, University of Michigan, Ann Arbor, Michigan, USA; 8 National Health Research Authority and Public Health, Lusaka Apex Medical University, Lusaka, Zambia; 9 Health Research Methods, Evidence and Impact, McMaster University, Hamilton, Ontario, Canada

**Keywords:** COVID-19, vaccines, health systems, health policy

**Summary box**

- Development of vaccines is a major breakthrough in the fight against the SARS-CoV-2 virus.
- Much attention has been paid to how to prioritise between patient groups for vaccination and how to ensure equity, especially in low-income countries, but there are other important decisions that need to be made.
- These decisions include: (a) choosing between the various vaccines that will become available, (b) continuing to invest in other aspects of the COVID-19 response and (c) balancing the COVID-19 response with the need to invest in other healthcare that has suffered during the pandemic.
- Although these decisions are inherently difficult, principles of good priority setting can be helpful; these principles include: evidence-based and transparent decision-making, participation of stakeholders and a focus on the implementation of decisions.

## Introduction

Successful vaccination against the SARS-CoV-2 virus is a decisive development in the international response to the pandemic. It also has substantial implications for how governments and international bodies deploy their resources, as major decisions will need to be made in a fast-moving and uncertain environment. Current trends in vaccine development have stimulated much commentary on how vaccines deemed to be the safest and most effective should be allocated, both at the global level (to ensure access for low-income countries)^1 2^ and within countries (to prioritise critical personnel and the most vulnerable population groups).^3^ We recognise the importance of mass vaccination as a public health measure, and the crucial need to promote equity and solidarity across countries.^4 5^ We also recognise that prioritisation is required *within* countries so that resources are directed to best protect life, reduce inequities and increase public confidence. However, based on analysis of the COVID-19 response so far, we would argue that at least three additional forms of prioritisation are required: between vaccines; between vaccines and other elements of the pandemic response; and between COVID-19 and other areas of health provision. In each case, decision makers should concentrate as much on infrastructure and implementation as on principles of resource allocation.

## Selecting vaccines: the long term

As we have already seen, there is a temptation for governments to invest most of their available resources in the vaccines exhibiting the greatest promise of efficacy and safety. However, there are many alternative vaccines currently undergoing phase III clinical trials. If the objective is to reduce the global burden of COVID-19 in the long term and avoid deepening inequities in the process, both vaccine cost-effectiveness and equity must be considered.

Asssessing cost-effectiveness between vaccines is hampered by their independent development and evaluation to date. Head-to-head comparisons may be further compromised by the need to account for variations in effectiveness and their ability to protect different subpopulations. Ultimately, implementation costs are likely to prove to be as, if not more, important than narrow cost-effectiveness profiles in determining which options are best suited in any given context. Cost analysis should therefore encompass logistical and distribution issues, which are likely to be significant drivers of affordability and influenced by, for example, the requirement for vaccine storage at very low temperatures.^6^

Vaccine costs and cost-effectiveness should be considered in the wider context of equity of access and outcome, and fair allocation of resources within and across populations.^7 8^ Though COVID-19 vaccines may initially be funded by donors, all countries will need long-term, mass COVID-19 vaccination strategies to ensure equitable and sustainable practices. Circumstances in which access is dependent on out-of-pocket contributions, or where available vaccines are perceived as unsafe and/or ineffective, will fuel inequity, antivaccine propaganda, and black and grey vaccine markets.^9^

## Prioritising across the COVID-19 response

The experience of COVID-19 so far has been marked by variation in system responses and notable resource shortages for both public health and treatment measures.^10^ The cost of investing in mass vaccination should therefore be weighed against the continued need for these other public health and treatment measures, as the vaccine will not immediately eradicate the virus. Embedding vaccination programmes in a well-resourced overall pandemic strategy and infrastructure will help with roll-out. Contact tracing, protective equipment, treatment and environmental mitigation strategies such as indoor ventilation will all still be required, although in (hopefully) reducing orders of magnitude. These tools will continue to play a critical role in the pandemic response in contexts where vaccines are not available for whole populations at once, or vaccine effectiveness diminishes. Recent reports of virus mutations in mink in Denmark and other countries and its potential spread to humans highlight the importance of ongoing vigilance and public health investment across the COVID-19 response.^11^

## Prioritising across healthcare programmes

An accompanying feature of the pandemic has been the suspension of many areas of routine care, including vaccination for preventable diseases.^12 13^ The ramifications of this will have to be dealt with not only in the months ahead but for many years to come. Backlogs and capacity constraints will result in excess mortality attributable to COVID-19—already modelled for several disease areas such as cancer^14 15^ and cardiovascular disease.^16 17^ Catching up on backlogs will place additional, competing demands on the resources available. Investment in vaccination will need to account for this ‘suspended care need’ and should be weighed against the immediate and longer term requirements for the delivery of routine health programmes.

In lower income countries (LICs), the consequences are especially stark, with lives lost due to a lack of routine urgent care through unemployment, restrictions on movement and healthcare, as well as a wider reluctance to attend healthcare facilities due to fears of contracting COVID-19. Furthermore, previous health emergencies, such as the most recent Ebola outbreak in West Africa, have shown a decline in funding for other essential programmes such as child and maternal health services.^18^ The impact of further diverting scarce resources away from other areas of need will therefore need to be closely monitored and evaluated in order to understand the potential consequences of placing a higher value on one life saved from COVID-19 over one life saved from other conditions.

## Focus on implementation

Our analysis of COVID-19 international pandemic responses highlights the critical importance of planning for effective implementation of any mass vaccination programme.^19^ If the global community is seriously committed to a fair and just international COVID-19 vaccination strategy, the conversation must go beyond mitigating inequities in nations’ purchasing power and access to safe and effective vaccines. There are additional inequities across and within countries in terms of the resources available for mobilisation and infrastructure in place to support a mass vaccination campaign.^20^ Meanwhile, the burden on agencies at the forefront of the COVID-19 response cannot be overstated. Vaccine roll-out, however welcome, is an additional strain not only in LICs but also increasingly in high-income countries.^21 22^ There is a need to support health systems to sustain routine services—this is more critical for weaker health systems seeking to prevent excessive avoidable mortality.^20^ Lastly, as countries plan to roll out COVID-19 vaccination, it is important that effective strategies to deal with vaccine hesitancy are developed and implemented.^23 24^

## Conclusions

The fundamental tenets of good priority setting include: taking an explicit approach to decision-making; meaningful stakeholder engagement; consideration of public values, ensuring mechanisms for appeals and revisions; and use of relevant evidence.^25 26^ Furthermore, for priority setting to be impactful, resource allocation should be aligned with set priorities.^25 27^ In the case of vaccination against COVID-19, such tenets will be crucial, as mismanaged priority setting would have disastrous consequences for health, equity and trust in public health and policymakers. However, the challenges extend beyond choosing which patient groups to prioritise, and there is a risk that a narrow focus on such questions will distract from other choices that need to be made.

When selecting between vaccines for investment, governments should balance the requirement for urgency with the importance of ongoing evidence-informed decision-making, as more becomes known about vaccine characteristics, adverse effects, duration of immunity and extent of protection from transmission. In evaluating vaccine options, decision makers should go beyond narrow cost-effectiveness profiling to assess implementation costs, especially those that are likely to vary across vaccines. Taking a ‘real world’ approach to priority setting requires decision makers to accept uncertainty with respect to the unfolding context, and conflicting ethical imperatives both in policy and wider society. There are no simple decision rules that can be transferred across contexts^28^ and consistency will best be served by the building of ‘case law’ as a means to inform, defend and refine decisions, and enable constant and careful monitoring and evaluation to ensure the greatest impact.^29^

In these conditions of uncertainty and occasional conflict, the design of vaccination programmes cannot be a purely technical task, and hence, there is a need for greater participation in decision-making than has been exercised so far with, for example, poor gender and racial representation on COVID-19 planning task forces.^30^ Successful vaccination programmes will require buy-in from patient groups, local implementers and healthcare professionals (including the largely unpaid community health workers who will be relied on to support vaccination programmes in LICs), as well as major players from industry and global donors. Most crucially, investment in COVID-19 vaccination should not be pursued at the expense of ongoing public health prevention and other health needs.

Health equity as an over-riding aim of the global COVID-19 response requires that priority setting goes beyond decision-making for vaccines and takes a more holistic view that also considers the challenges associated with the long-term effects of vaccine selection, priority setting across COVID-19 response and healthcare programmes as well as implementation.
